# Polysaccharides as Carriers of Polyphenols: Comparison of Freeze-Drying and Spray-Drying as Encapsulation Techniques

**DOI:** 10.3390/molecules27165069

**Published:** 2022-08-09

**Authors:** Ivana Buljeta, Anita Pichler, Josip Šimunović, Mirela Kopjar

**Affiliations:** 1 Faculty of Food Technology Osijek, Josip Juraj Strossmayer University of Osijek, F. Kuhača 18, 31000 Osijek, Croatia; 2 Department of Food, Bioprocessing and Nutrition Sciences, North Carolina State University, Raleigh, NC 27695, USA

**Keywords:** polysaccharides, polyphenols, freeze-drying, spray-drying

## Abstract

Polyphenols have received great attention as important phytochemicals beneficial for human health. They have a protective effect against cardiovascular disease, obesity, cancer and diabetes. The utilization of polyphenols as natural antioxidants, functional ingredients and supplements is limited due to their low stability caused by environmental and processing conditions, such as heat, light, oxygen, pH, enzymes and so forth. These disadvantages are overcome by the encapsulation of polyphenols by different methods in the presence of polyphenolic carriers. Different encapsulation technologies have been established with the purpose of decreasing polyphenol sensitivity and the creation of more efficient delivery systems. Among them, spray-drying and freeze-drying are the most common methods for polyphenol encapsulation. This review will provide an overview of scientific studies in which polyphenols from different sources were encapsulated using these two drying methods, as well as the impact of different polysaccharides used as carriers for encapsulation.

## 1. Introduction

Polyphenols are secondary plant metabolites consisting of an aromatic ring to which one or more hydroxyl groups are attached [1]. These compounds are synthesized through plant development and/or as a plant’s response to environmental stress conditions [2]. Even when they are at low concentrations in plants, polyphenols protect them from predators or ultraviolet damage [3]. They are known as natural antioxidants and therefore have many beneficial effects on health (e.g., antimicrobial, anti-inflammatory, antioxidant effect, etc.) [4,5,6]. The health benefits of polyphenols are influenced by the matrix in which they are processed and ultimately consumed [7]. One well-known property of polyphenols is the positive influence on diabetes and obesity due to the possibility of inhibition of digestive enzymes such as α-glucosidase and α-amylase [8]. Anthocyanins are a group of polyphenols responsible for the red, blue and purple color of fruit and vegetables. The major anthocyanin found in most plants is cyanidin-3-glucoside, correlated with reduced reactive oxygen species (ROS) levels and antioxidant potential in in vitro conditions [9,10]. Flavan-3-ols are a subgroup of flavonoids and their main representatives are catechin, epicatechin, epigallocatechin and epigallocatechin-3-gallate. These compounds are abundantly found in green tea, strawberries and black grapes. Studies showed a positive effect of catechin in Alzheimer’s and Parkinson’s diseases, diabetes and in cancer treatment [11]. Gallic acid, a polyphenol from a group of phenolic acids, a subgroup of hydroxybenzoic acids was the subject of many studies that have proven its significant antioxidant, anticarcinogenic, antimicrobial and antimutagenic effects [12].

Due to the presence of unsaturated bonds in their structures, polyphenols are sensitive to various environmental conditions such as the presence of oxygen, light and water [2]. The presence of water is the most important factor, due to its essentiality in most chemical reactions [13,14]. In the food industry, thermal processes are mostly used to obtain edible, microbiologically safe foods, to improve digestibility, and to modulate their textures, flavors and colors [14]. During these processes, structural changes occur leading to the degradation of polyphenols which are often ignored [14]. In order to maintain their stability, they need to be protected, and one of the possible ways is encapsulation in which polysaccharides, proteins, lipids or combinations thereof can be utilized as carriers. In that way, the preservation of polyphenolic properties is achieved over longer periods because the carrier materials represent a barrier to oxygen and water [2]. By encapsulation of polyphenols, besides increased stability, mitigation of unpleasant tastes or flavors, controlled release, improved aqueous solubility and bioavailability can be achieved [3]. Drying has effect on the material’s appearance and chemical composition. It also prolongs shelf life and inhibits enzymatic degradation and microbial growth of materials or foods [15]. Adequate selection of a drying method and operating conditions yields foods with slight changes in appearance and maximum retention of bioactive compounds [15].

Spray-drying is a commonly used method for encapsulating due to its simple regulation and control, limited cost, and continuous operation [16]. Freeze-drying is also often used for encapsulation of thermosensitive compounds and materials with some disadvantages such as higher unit cost and long processing time [17]. Suitable selection of carrier and encapsulation technique leads to successful incorporation and retention of bioactive compounds [2]. Food enriched with encapsulated polyphenols can be a versatile and cost-effective approach [14]. In addition, this approach enables other features such as controlled release, improved bioaccessibility and bioavailability for absorption [14].

This paper will provide the literature review of spray-drying and freeze-drying for the encapsulation of polyphenols from different sources. Moreover, with emphasis on polysaccharides, the influence of carrier materials on polyphenol encapsulation will be reviewed.

## 2. Polyphenols

**Figure 1:**
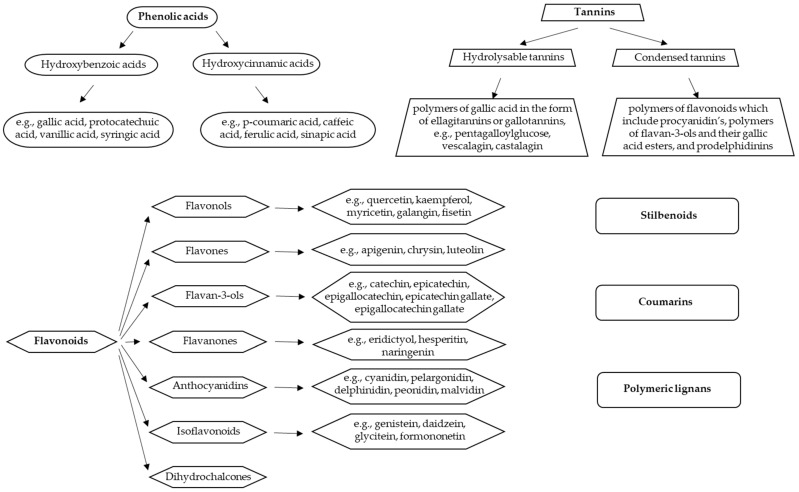
Classification of polyphenols and the most important representatives of individual groups (adapted from Dobson et al. [18]).

Polyphenols include various different compounds divided into several classes: phenolic acids, flavonoids, stilbenoids, tannins, coumarins, and polymeric lignans (Figure 1) [18]. Their chemical structure may vary from simple to complex. The largest group of polyphenols is that of flavonoids, divided into several subgroups (flavonols, flavones, flavanols, flavanones, anthocyanidins, isoflavonoids, and chalcones) [19]. Their structure consists of two phenyl groups linked with a three-carbon bridge. According to the degree of oxidation and unsaturation of the three-carbon segment, they differ from each other. Different sugar molecules can be attached to the hydroxyl groups of flavonoids. They are usually in a glycosidic form which improves their solubility in water. Acylation of the glycosides where sugar hydroxyls are derivatized with acid (such as ferulic and acetic acids) is also common. The interconnection between several basic units of polyphenols makes larger and more complex structures, such as hydrolysable tannins and condensed tannins [20]. Phenolic acids represent a large group of hydrophilic polyphenols and they are constituted of a single phenyl ring [14]. Due to the diversity in the structures of polyphenols, they possess different properties (such as solubility and polarity) [20].

Polyphenols exist ubiquitously in vegetables and fruits and their consummation is very desirable [14]. Various positive bioactivities of polyphenols toward pathologic conditions are known, through their antioxidant properties. These molecules are capable of donating hydrogen atoms and electrons [14]. One such activity is the anticancer property of polyphenols [21]. Hollman et al. [22] reviewed the antioxidant activities of polyphenols within the organism and their positive impact on cardiovascular health. In addition, polyphenols contribute to the sensory quality of the products (wine, jellies, juices, chocolate, etc.). They affect color, bitterness, turbidity, etc. [6,23]. Due to their many functional properties, polyphenols are of great interest to the food, chemical and pharmaceutical industries [2].

## 3. Encapsulation

**Figure 2:**
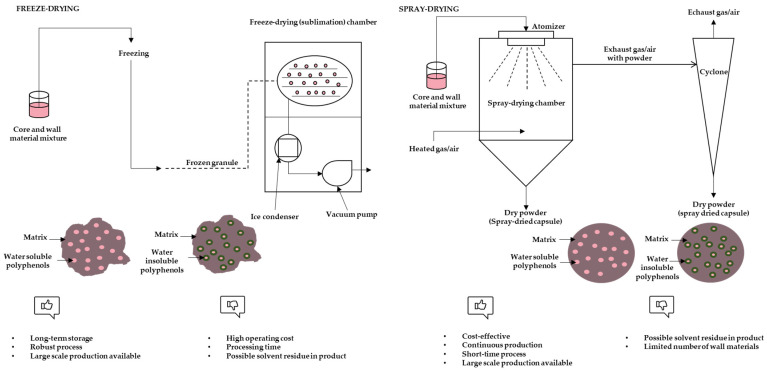
Schematic view of freeze-drying and spray-drying process, characteristics of particles and advantages and disadvantages of methods (adapted from Fang and Bhandari, [25]; Grgić et al., [26]).

Encapsulation is the process of entrapment of active compounds into particles to enable isolation or controlled release of given compounds. The main role of encapsulation is the protection of sensitive active compounds from degradation [17]. Various techniques can be used for encapsulation depending on core material, size of required particles, physical state, or sensitivity to high temperature [24]. These techniques include freeze-drying, spray-draying, extrusion, fluidized bed coating, spray-cooling/chilling, coacervation, liposome entrapment, cocrystallization, vacuum-drying, centrifugal suspension separation, nanoencapsulation, molecular inclusion and emulsification [13]. Encapsulation with coacervation is based on phase separation of a hydrocolloid from an initial solution and subsequent deposition of the formed coacervate phase around an active ingredient suspended in media. It is considered an expensive technique but very beneficial for high-value compounds [25]. Extrusion is a process based on passing a polymer solution with active ingredients through a nozzle (or syringes) into a gelling solution. Usually the used wall material is sodium alginate while calcium chloride solution serves for capsule forming. It is easy to execute on a laboratory scale with long shelf-life capsules, while scale-up of this technique is expensive and demanding with a limited choice of wall materials [26]. Emulsification includes the dispersion of one liquid into the other (two immiscible liquids) in the form of droplets. An emulsifier is required for stabilization and by application of drying, a powder form of encapsulates can be achieved [26]. The molecular inclusion method is also known as host-guest complexation. Apolar guest molecules are trapped inside the apolar cavity of host molecules (such as cyclodextrins) through non-covalent bonds [26]. Cocrystallization techniques include modification of the crystalline structure of sucrose to an irregular agglomerated crystal with porous structure in which active ingredient can be incorporated [25]. Fluidized bed coating is also referred to as fluidized bed processing, air suspension coating, or spray coating. The principle is that coating is applied to the particles suspended in the air. For this technique a wide range of coating materials such as aqueous solutions of cellulose, starch derivates, gums, or proteins is suitable [27]. Spray chilling is also known as spray cooling, prilling, or spray congealing. The basic principle is similar to spray drying with the key difference of using a cooling chamber instead of a drying chamber. Regarding coating materials, only lipid-based materials (fats, waxes, fatty acids, fatty alcohols and polyethylene glycols) are used [27]. Nanoencapsulation is an innovative trend in the field of food technologies. The final results are particles of diameters ranging from 1 to 1000 nm. The term nanoparticles includes nanospheres and nanocapsules. The first one has a matrix-type structure where the active ingredients can be adsorbed at the sphere surface or encapsulated in particles. In nanocapsules, the active ingredient is limited to a cavity with an inner liquid core surrounded by a polymeric membrane. Nanoparticles have a larger surface area, increased solubility, enhanced bioavailability and improved controlled release [25]. Freeze-drying and spray-drying are the most frequently employed methods for removing water from foods with encapsulation effects [28]. A schematic view of these methods is presented in Figure 2.

The wide range of polyphenol biological activities can be restricted due to their low stability, low bioavailability, and unpleasant flavor [14]. Encapsulation of polyphenols improves their stability during storage and can also be used to achieve the masking of unpleasant flavors in foods (such as bitter taste and astringency) [2]. Food with high intensity of bitterness and astringency elicit negative consumer reactions. Compounds responsible for that are flavanols and flavonols. Flavan-3-ol monomers such as catechin, epicatechin, epigallocatechin, epicatechin gallate and epigallocatechin gallate as well as their oligomers proanthocyanidins (condensed tannins) are abundant in wine and tea. It can be said that flavanols are the main compounds that cause bitterness and astringency in tea and red wine (with the exception of caffeine in tea) [29]. The method selected for encapsulation must be based on the polyphenol’s characteristics such as chemical structure, thermophysical stability, solubility, affinity to coating material, target properties such as particle size and morphology, among others [14].

### 3.1. Freeze-Drying

Freeze-drying, also known as lyophilization, is a drying technique based on the phenomenon of sublimation. This technique allows the long-term preservation of heat-sensitive and oxidation-prone compounds, as well as foods and other biological materials, since it is conducted at low temperatures and under vacuum [30]. This method does have disadvantages such as higher unit cost and processing time [17]. Despite those issues, freeze-drying is widely used to obtain high-value food products and is considered a standard method for encapsulation in most research studies [30].

The freeze-drying process includes the complete freezing of samples, ice sublimation (primary drying) and desorption of remaining unfrozen/bond water (secondary drying). In the first step, the freezing rate determines the formation and size of ice crystals. Large ice crystals formed by the slow rate of freezing can sublimate easily and increase the primary drying rate. In primary drying, the shelf temperature will increase using a vacuum to start the sublimation. It is necessary that product temperature is 2–3 °C below the temperature level of collapse, at which the product can lose its macroscopic structure. The endpoint of the primary drying phase is a key parameter to determine because the increased temperature (in the secondary drying) before the sublimation of all ice could collapse the final product quality [30].

### 3.2. Spray-Drying

In the food industry spray-drying is the most frequently used technique. It is economical, flexible and can be used continuously with easy scale-up [13]. Advantages over the other methods are higher effectiveness and shorter drying time [16]. This technique is based on transforming a material from liquid form into powder form [24]. However, when spray-drying is used for polyphenol encapsulation, conditions must be optimized in order to avoid an accelerated degradation [17]. Depending on process conditions and formulations, spray-drying micrometric capsules can be core-and-shell, multiple core or matrix type [14]. Before spray-drying, a solution or suspension of polyphenols with carriers must be obtained, followed by atomizing into a hot air stream.

### 3.3. Particle Morphology

Spray-dried particles are usually spherical with varying diameters and concavities, regardless of the choice of coating material [17]. Mean size range of such particles range from 10 µm to 100 µm [25]. The formation of concavities is associated with shrinkage of the particles due to dramatic loss of moisture after cooling. On the other hand, powders obtained by freeze-drying have a flake-like structure or one that resemble broken glass. The reason could be the low temperature of the process which results in the absence of forces to break the frozen liquid into droplets. Differences in surface morphology of freeze-dried powders could be due to different coating agents. For example, powder with soybean protein and maltodextrin as coating materials had a spherical porous structure, while powders with only maltodextrin lost their porous structure [17]. The final particle size of freeze-dried powders depends on the grinding procedure, not on the drying process [31].

### 3.4. Polyphenol Carriers

Materials used for encapsulation, which makes a protective shell, should be biodegradable and food-grade. They also must be able to establish a barrier between an internal phase and its surroundings [13]. Selection of coating material influences encapsulation efficiency and encapsulates stability [24]. Commonly used materials are carbohydrates such as maltodextrin, cyclodextrins, gum Arabic and modified starch. These materials lead to an increase in the glass transition temperature of the dried product. By trapping a bioactive compound, they enable its preservation against stickiness, temperature, enzymatic and chemical changes [13].

Maltodextrin is one of the most often used carriers, having a low bulk density and viscosity and high solubility at high solids contents [16,24]. It is obtained by partial hydrolysis of starch using an enzyme or acid. Maltodextrin has the ability to retain volatile compounds. Its disadvantages, such as low emulsibility, are often overcome by combining maltodextrin with other materials [16].

Cyclodextrins are safe for food applications and broadly studied as hosts for encapsulation [3]. The commonly used cyclodextrins are α-, β- and γ-cyclodextrin. They possess a hydrophobic central cavity and a hydrophilic external part [32]. Due to this structure, guest molecules (different organic and inorganic molecules) are able to be accommodated into the cavity. The hydrophilic external surface provides aqueous solubility [3,24]. The application of cyclodextrins in spray-drying is limited due to their low water solubility (1.8%). This limitation can be overcome by cyclodextrin modification. An example of such modification is hydroxypropyl-β-cyclodextrin which has a higher water solubility (60%) and thus can be subjected to drying techniques [24].

Gum Arabic consists of galactose, rhamnose, arabinose, 4-O-methyglucuronic acid and glucuronic acid and its natural source is the acacia plant (stems and branches). Furthermore, its low viscosity and high solubility, stable emulsions and high retention rates of volatile compounds have enabled a broad range of applications of this polymer [16]. On the other hand, some disadvantages such as low production yield and a consequently higher price make it less accessible [33].

## 4. Application of Spray-Drying and Freeze-Drying for Encapsulation of Polyphenols

During freeze-drying of foods rich in polyphenols, cells are disrupted, and therefore exposed to an increased enzyme activity (polyphenol oxidase and peroxidase enzyme) upon thawing, and the degradation of the polyphenols can occur [34,35]. However, some studies have shown that the amount of polyphenols may increase after this process. The flavonol content in freeze-dried onions increased, which can be attributed to the release of polyphenols from the matrix [36]. Wilkowska et al. [24] observed that freeze-dried powders had 1.5 times higher retention of anthocyanins than spray-dried ones. From studying the values of total polyphenols content, it has been noticed that when applying spray-drying, 73% of compounds were lost. Encapsulates of polyphenols in coffee grounds extract achieved by freeze-drying and spray-drying with maltodextrin, gum Arabic and maltodextrin:gum Arabic (1:1) as carrier materials were evaluated for total polyphenols content and flavonoid content. The results showed that freeze-drying was a more effective technique for retention of polyphenols and flavonoids and maltodextrin a more efficient carrier. On the other hand, spray-dried particles possessed a higher antioxidant activity than freeze-dried ones [2]. One interesting investigation was conducted on developing novel protein ingredients fortified with blackcurrant concentrate. As a source of protein, they used whey protein isolate and freeze-drying and spray-drying techniques. Encapsulates obtained by spray-drying possessed a higher total polyphenols content, anthocyanins content and encapsulation efficiency compared to the freeze-dried ones [28]. Robert et al. [37] encapsulated polyphenols from pomegranate juice and ethanolic extract by spray-drying, and observed a higher encapsulation efficiency when soy protein isolates were used compared to the maltodextrin. On the other hand, capsules with maltodextrin, stored at 60 °C in an oven for 56 days, resulted in a lower degradation of polyphenols and anthocyanins. Wu et al. [38] investigated the physicochemical properties and nutritional characteristics of functional cookies with incorporated encapsulated blackcurrant polyphenols. For the preparation of encapsulates, whey protein isolate and blackcurrant concentrate were used and the applied encapsulating techniques used were freeze-drying and spray-drying. The results of total polyphenols content were higher for enriched cookies with freeze-dried encapsulates than for those with spray-dried encapsulates.

Ersus and Yurdagel [39] observed that during spray-drying, maltodextrins with higher DE (equivalents of dextrose) are more sensitive to higher outlet air temperatures. Heating could lead to structural deformations due to shorter chains and oxidation of free glucose functional groups at the open ends. Their results confirmed the encapsulation of black carrot anthocyanins using maltodextrin DE 20-21 for 20% feed solid content and 160–180 °C drying temperatures. Gomes et al. [40] obtained a higher retention of papaya pulp polyphenol and flavonoid compounds in spray-dried products than in freeze-dried products. Vanillic acid had an enormous decrease of 76% after freeze-drying. Enzymatic reactions with the action of peroxidase and polyphenol oxidase are likely to occur in the freeze-drying process. Moreover, the disrupted material structure caused by the formation of ice crystals and lower exposure to oxygen can cause the liberation of these enzymes. Furthermore, artepillin C concentration increased three times after drying, which can lead to the false-positive results of the spray-drying technique. It is known that processes at high temperatures may release more bound polyphenols which cannot be detected in fresh samples [40]. Saikia et al. [41] encapsulated polyphenols from *Averrhoa carambola* pomace using maltodextrin and freeze-drying and spray-drying methods. The authors obtained a higher encapsulation efficiency in freeze-dried encapsulates. It might be that some polyphenols are destroyed during spray-drying, due to their sensitivity to heat. During the spray-drying process, fine misty droplets with increased surface are obtained and due to that higher surface, they are more exposed to heat. Moreover, during atomization, some amount of carrier can be eliminated from the core material and the partially covered capsules thus obtained can be destroyed by heat. During the freeze-drying process, no atomization or heat exposure are present. These authors also observed a decrease in surface polyphenols with higher maltodextrin content [41]. On the other hand, high inlet temperatures applied in spray-drying are short-lived, so this technique is less destructive for bioactive compounds compared to the other conventional thermal processes [42]. It has to be taken into consideration that in the freeze-drying process, obtained powders are ground and this process increases the possibility of contact with air, resulting in oxidation reactions [42,43]. In the freeze-drying process, the formation of a sawdust-like form is usual, leading to a lower surface area/volume ratio. Additionally, by the spray-drying process, smaller sized microspheres with a larger surface area were obtained using the spray-drying process (for the same amount of material as for freeze-drying), which led to the deterioration of the surface polyphenols [2]. Considering the short time of exposure to high temperatures during spray-drying, this technique seems good for the encapsulation of polyphenols. Dealing with thermosensitive and highly valuable materials, freeze-drying is a suitable method [33]. Table 1, Table 2 and Table 3 present the studies on the encapsulation of polyphenols using freeze-drying and spray-drying techniques.

## 5. Application of Encapsulated Polyphenols in Food Products

Due to the increasing awareness of consumers toward health and the consumption of food that promotes health, the enrichment of food products with encapsulated polyphenols and replacement of artificial food additives with natural ones is strongly supported [78]. Over the last few years, applying encapsulated polyphenols in food products has been on the rise. In reviewing scientific papers, those dealing with encapsulated polyphenols with polysaccharides were singled out.

Yogurt has high water content and a low pH value which makes it challenging to incorporate polyphenols with poor solubility. Encapsulated polyphenols into hydrophilic wall materials can overcome these shortcomings [78]. Robert et al. [37] encapsulated polyphenols from pomegranate with maltodextrin and soybean protein isolates and incorporated them in yogurt. Encapsulates with maltodextrin had the lower degradation rate during storage. Moreover, mushroom extract rich in polyphenols, encapsulated with maltodextrin crosslinked with citric acid, was incorporated in yogurt [79]. In a study of El-Messery et al. [77], polyphenols extracted from apple peel were encapsulated with maltodextrin, whey protein and gum Arabic using spray-drying and freeze-drying. The obtained powders were used in supplementing yogurt. Results showed no significant influence of powders on the physiochemical and texture properties of samples. The authors suggested that those encapsulated polyphenols can be used as a functional food ingredient for yogurt. One interesting study dealt with encapsulation of eugenol-rich clove extract in maltodextrin and gum Arabic by spray-drying. These encapsulates were incorporated into soybean oil for increasing antioxidant activity. Potatoes fried in that oil had better sensorial properties than ones fried in oil with butylated hydroxytoluene (synthetic antioxidant) [80]. Encapsulated polyphenols can also be incorporated into bread. Ezhilarasi et al. [81] enriched bread with encapsulated *Garcinia* fruit polyphenols with maltodextrin and whey protein isolates. Furthermore, green tea polyphenols, encapsulated using β-cyclodextrin and maltodextrin by freeze-drying and spray-drying, were added to bread. Bread quality (volume and crumb firmness) didn’t change compared to the control sample [82]. Furthermore, anthocyanins from red onion skins were encapsulated using gum Arabic, soy protein isolate and carboxymethyl cellulose as wall materials, and applying the gelation and freeze-drying techniques. The powder, which has the highest encapsulation efficiency, has been added to crackers and results showed improved antioxidant activity of these enriched crackers. The authors suggest the suitability of applying such additives in bakery products [83]. Table 4 presents some other studies that dealt with incorporating encapsulated polyphenols with polysaccharides using freeze-drying and spray-drying techniques into food products.

## 6. Conclusions

Bioactive compounds, such as polyphenols attract a lot of attention from scientists, functional food product developers and consumers due to their health-promoting effects. Most of these compounds are chemically unstable and encapsulation techniques have been widely applied in order to enhance their stability. Nevertheless, freeze-drying is still assumed to be the most suitable for heat-sensitive compounds. The adequate method of encapsulation depends on the type of polyphenol and material for encapsulation, however. Therefore, it cannot be generally said that freeze-drying is better than spray-drying or vice versa. Choosing a polyphenol carrier is important in order to achieve effective encapsulation. While freeze-drying will definitely result in a higher quality and better bioactivity retention compared with conventional spray drying methods, its application is excessively expensive, time consuming and limited in throughput capacity for most commercial applications. Therefore, in order to increase the number and variety of products on the consumer markets, as well as the application range of encapsulated polyphenols, new technologies will need to be researched and if necessary developed.

This paper contributes to the insights into previously researched and optimized encapsulating conditions for a large number of polyphenol-rich materials.

## Figures and Tables

**Table 1:** Studies of spray-drying application for encapsulation of polyphenols from different sources.

Bioactive Material		Reference
Roselle (*Hibiscus sabdariffa* L.) extract		[44]
Carrier	Maltodextrin, gelatin, pectin, carboxymethyl cellulose, carrageenan, gum Arabic and whey protein	
Conditions	0.5 mm—nozzle diameter; 400 kPa—compressor air pressure; 7.5 mL/min—feed flow rate; 155 °C—inlet temperature; 55 °C—outlet temperature; 56 m^3^/h—air flow rate	
Observations	Pectin can be a suitable carrier of roselle polyphenols because it retained the highest amount of polyphenols (98.20 mg/100 mg)	
Plum (*Prunus salicina* Lindl.) polyphenols		[45]
Carrier	Maltodextrin, gum Arabic, gelatin, chitosan and β-cyclodextrin	
Conditions	150 °C—inlet temperature; 85 °C—outlet temperature; 850 mL/h—feed flow rate; 90 MPa—atomization pressure; 1.15 m^3^/min—blower rate; 0.7 mm—nozzle diameter	
Observations	Powders obtained with maltodextrin/chitosan possessed the highest stability as well as retention of total polyphenols (94%) after storage (60 days at 25 °C)	
Pomegranate juice anthocyanins		[46]
Carrier	Gum Arabic, modified starch from waxy maize and maltodextrin	
Conditions	1 mm—nozzle diameter; 1 kg/h—mass flow rate; 162–170 °C—inlet temperatures; 89–93 °C—outlet temperatures; 500 m^3^/h—air flow rate	
Observations	By using gum Arabic:modified starch from waxy maize (1:1) mixture, up to 70% of total monomeric anthocyanins were retained	
Cinnamon (*Cinnamomum zeylanicum*) proanthocyanidins		[47]
Carrier	Maltodextrin	
Conditions	1.2 mm—nozzle diameter; 130 °C and 160 °C—drying temperatures; 40 mL/min—feed flow rate	
Observations	The highest retention of proanthocyanidins (100%) was obtained in the sample with 20% of maltodextrin and dried at 160 °C; during 90 days of storage, maltodextrin contributed to stability of proanthocyanidins	
Polyphenols of *Orthosiphon stamineus* leaves		[48]
Carrier	Maltodextrin and whey protein isolate	
Conditions	0.5 mm—atomizer; 180 °C—inlet temperature; 407 mL/h—feed flow rate	
Observations	By using 5.33 wt.% of maltodextrin, the highest retention of sinensetin (82.24%), rosmarinic acid (82.67%) and eupatorine (80.19%) was obtained	
*Fadogia ancylantha, Tussilago farfara* and *Melissa officinalis* extracts		[49]
Carrier	Maltodextrin and apple pectin	
Conditions	120 °C—inlet temperature; 69–71 °C—outlet temperature; 5 mL/min—feed flow rate; 0.5 mm—nozzle diameter; 500 L/h—drying air flow; 6 bar—air pressure; 100%—aspiration	
Observations	By using 10:1 maltodextrin:pectin ratio, 3% w/v of raw dried extract was encapsulated; loading efficiency values were very high: 80.1%, 91.5% and 97.2% for Tussilago, Melissa and Fadogia polyphenols, respectively	
Black carrot (*Daucus carota* L.)		[39]
Carrier	Maltodextrins (DE 10, DE 28–31 and DE 20–23)	
Conditions	160 °C, 180 °C and 200 °C—inlet temperatures; 107 °C, 118 °C and 131 °C—outlet temperatures; 5 mL/min—feed flow rate	
Observations	Powders with maltodextrin DE 20–23 contained the highest anthocyanin concentration (630 mg anthocyanin/100 g dry matter of powder)	
Mountain tea (*Sideritis stricta*) extract		[50]
Carrier	β-cyclodextrin, maltodextrin and gum Arabic	
Conditions	145 °C, 155 °C and 165 °C—inlet temperatures; 75 °C—outlet temperature; 500 L/h—flow rate; 70%—aspiration rate; 240–640 mL/h—feed rate	
Observations	By increasing inlet air temperature from 145 °C to 155 °C, a 4% increase in total polyphenols content occurred, but a further increase in inlet temperature had a negative effect; an increase in the concentration of carriers resulted in a decrease in total polyphenols content (18.96 g/100 g—without carrier, 7.61 g/100 g—for 3 g/100 g of carrier, and 5.43 g/100 g—for 5 g/100 g of carrier)	
Cactus pear (*Opuntia ficus-indica*)		[51]
Carrier	Maltodextrin and inulin	
Conditions	140–160 °C and 120–160 °C—inlet temperatures for maltodextrin and inulin, respectively; 600 L/h—air flow; 10 mL/min—feed rate; 20 psi—atomization pressure	
Observations	Optimal conditions for encapsulation with maltodextrin were 3:1 ratio of core/coating material and 140 °C inlet temperature while for inulin the optimal ratio was 3:1 for encapsulation of pulp extract and 5:1 for encapsulation of ethanolic extract and 120 °C inlet temperature; concentration of polyphenols after encapsulation ranged as follows: cactus ethanolic extract + inulin (2410 mg GAE/g powder) > cactus pulp + maltodextrin (2135 mg GAE/g powder) > cactus pulp + inulin (2028 mg GAE/g powder) > cactus ethanolic extract + maltodextrin (1812 mg GAE/g powder)	
Açai (*Euterpe oleracea* Mart.) juice		[52]
Carrier	Maltodextrin (DE 10 and DE 20), gum Arabic, and tapioca starch	
Conditions	1.5 mm—nozzle diameter; 0.06 MPa—compressor air pressure; 15 g/min—feed flow rate; 140 °C—inlet temperature; 78 °C—outlet temperature; 73 m^3^/h—air flow rate	
Observations	Powders obtained with tapioca starch possessed the lowest anthocyanin content (3247.15 mg/100 g) while between the two types of maltodextrin (for DE 10 3436.85 mg/100 g and for DE 20 3402.30 mg/100 g) and gum Arabic (3415.96 mg/100 g) no statistical difference was determined in anthocyanin content	
Jaboticaba (*Myrciaria jaboticaba*) peel extracts		[53]
Carrier	Maltodextrin, gum Arabic, and modified starch from waxy maize	
Conditions	140 °C, 160 °C and 180 °C—inlet temperatures; 360 mL/h—feed flow rate; 20 mbar—vacuum; 28 m^3^/h—aspiration	
Observations	The highest anthocyanins retention was obtained at 160 °C air drying temperature using maltodextrin (99.02%) and gum Arabic/maltodextrin (100%) as carriers; a combination of maltodextrin with starch resulted in the lowest anthocyanins retention (around 80%) regardless of air drying temperatures	
Pomegranate (*Punica granatum*) polyphenols and anthocyanins of juice and ethanolic extracts		[37]
Carrier	Maltodextrin and soybean protein isolates	
Conditions	140–160 °C and 100–140 °C—inlet temperatures for maltodextrin and soybean protein isolates, respectively; 600 L/h—air flow; 10 mL/min—feed rate; 20 psi—atomization pressure	
Observations	Encapsulation efficiency of polyphenols was better in the soybean protein isolates matrix (76.2% for pomegranate juice and 82.9% for pomegranate ethanolic extract) than for maltodextrin (53.3% for pomegranate juice and 71.0% for pomegranate ethanolic extract); the highest encapsulation efficiency of anthocyanins from pomegranate ethanolic extract was in the soybean protein isolates matrix (100%) while the highest efficiency of pomegranate juice anthocyanins was in the maltodextrin matrix (86.6%)	
Rosemary (*Rosmarinus officinalis* L.) leaves polyphenols		[54]
Carrier	Maltodextrins (MDE 10 and 21), whey protein isolates and polyglycerol polyricinoleate	
Conditions	175 °C—inlet temperature; 90 °C—outlet temperature; 600 L/h—drying air flow; 18%—feed flow rate; 700 kPa compressor air pressure	
Observations	Higher encapsulation efficiency (around 42%) of total polyphenols was obtained when higher amounts of protein (4%) were used, pointing to the important impact of protein in that target delivery system; encapsulation efficiency with 2% of whey protein isolates was around 28%; type of maltodextrin showed no significant impact on encapsulation efficiency	
Epigallocatechin-3-gallate (EGCG)		[55]
Carrier	Gum Arabic and maltodextrin	
Conditions	160 °C—inlet temperature; 60 °C—outlet temperature	
Observations	Concentration of EGCG at surface was 8% and loading efficiency (EGCG in the inner space) was 85%	
Turmeric oleoresin (curcumin)		[56]
Carrier	Maltodextrin and gum Arabic	
Conditions	5 mL/min—feed flow rate; 150 °C, 175 °C and 200 °C—inlet air temperatures; 90 °C—outlet air temperature	
Observations	Gum Arabic used as a carrier at the inlet air temperature of 175 °C showed the highest encapsulation efficiency (71.74%) and total curcumin content (3.41 g/100 g)	
Barberry (*Berberis vulgaris*) extract		[57]
Carrier	Gum Arabic, gelatin and maltodextrin	
Conditions	800 mL/h—flow rate; 150 °C—inlet temperature; 100—outlet temperature	
Observations	Samples produced with gum Arabic and maltodextrin (core/wall material ratio 25%) in combination possessed the highest microencapsulation efficiency (96.22%)	
Roselle (*Hibiscus sabdariffa* L.) extract		[58]
Carrier	Maltodextrin and gum Arabic	
Conditions	180 °C—inlet air temperature; 80 °C—outlet air temperature; 12 mL/min—feed flow rate; 0.8 bar—atomizing-air pressure	
Observations	In sample where maltodextrin:gum Arabic ratio was 70:30, improved retention of polyphenols (465.80 mg GAE/100 g), anthocyanins (171.21 mg cyanidn-3-glucoside/100 g) and antioxidant activity (3.81 mmol Trolox/kg) was achieved	
Blackberry pulp		[59]
Carrier	Maltodextrin and gum Arabic	
Conditions	0.49 kg/h—flow rate; 145 °C—inlet temperature; 75–80 °C outlet temperature; 0.36 m^3^/h—drying air flow rate; 35 m^3^/h—aspirator flow rate	
Observations	Samples with maltodextrin and a combination of both carriers possessed higher anthocyanin retention (around 85%) than gum Arabic (around 78%)	
Chokeberry anthocyanins		[60]
Carrier	Maltodextrin, guar gum, gum Arabic, inulin, pectin and β-glucan	
Conditions	140 °C—drying temperature; 25%—pump flow; 600 L/h—air flow	
Observations	Capsules with β-glucan had the highest content of anthocyanins (first day and after 7 days of storage) while the ones with gum Arabic had the lowest content; the following encapsulation efficiency was observed: maltodextrin + gum Arabic—78.61%, maltodextrin + inulin—88.37%, maltodextrin + β-glucan—92.78%, maltodextrin + pectin—91.85% and maltodextrin + guar gum—92.98%	
Berries and roselle anthocyanins		[61]
Carrier	Gum Arabic, maltodextrin, whey protein isolate and agave fructans	
Conditions	180 °C—inlet temperature; 80 °C—outlet temperature; 13%—feed rate; 94%—air flow	
Observations	Retention of anthocyanins was 10.71–86.09%; retention of total polyphenols ranged from 34.71–100%; whey protein isolate was the best carrier agent for anthocyanins and phenolic compounds	

**Table 2:** Studies of freeze-drying application for encapsulation of polyphenols from different sources.

Bioactive Material		Reference
Red wine polyphenols		[62]
Carrier	Maltodextrin 20% DE 10	
Conditions	Freezing plate and condenser at −40 °C, vacuum below 200 µm Hg; process duration—40 h	
Observations	The freeze-drying process resulted with 97% polyphenol retention; there was no significant changes in polyphenols during 15 days of storage at 38 °C	
Fermented Miang wastewater bioactive compounds		[63]
Carrier	Maltodextrin, gum Arabic and modified starch	
Conditions	Samples were frozen at −18 °C for 24 h and then freeze-dried at −45 °C under a pressure of 0.133 mbar for 72 h	
Observations	Between used carriers, there were no statistical difference in total polyphenols content but in surface polyphenols content, differences were observed: by using gum Arabic in 10:1 core:coating material ratio (% *w*/*w*), the lowest concentration of surface polyphenols was observed with the highest encapsulation efficiency (98.05%); encapsulation efficiency for maltodextrin and modified starch was 89.07% and 81.58%, respectively	
Polyphenols of wastewater from Miang (fermented tea leaf) production		[64]
Carrier	Maltodextrin and gum Arabic	
Conditions	Samples were frozen at −18 °C for 24 h and then freeze-dried under a pressure of 0.133 mbar for 72 h	
Observations	The weight ratio of maltodextrin:gum Arabic mixture to concentrated fermented Miang water of 1:10 was the best for polyphenols encapsulation with 99.4% efficiency	
Cloudberry (*Rubus chamaemorus*) polyphenols		[65]
Carrier	Maltodextrins DE5–8 and DE18.5	
Conditions	Pressure < 0.1 mbar and duration of 48 h	
Observations	Maltodextrin DE 5–8 resulted in higher encapsulation efficiency for all polyphenols, especially for ellagitannins (99%), proanthocyanidins (94%) and flavonols (90%); the highest encapsulation efficiency values of maltodextrin DE 18.8 were for hydroxycinnamic acids and hydroxybenzoic acids (69% and 68%, respectively)	
Red onion peel polyphenols		[66]
Carrier	Maltodextrin and soybean protein isolate	
Conditions	Not defined	
Observations	The combination of maltodextrin with soybean protein isolate showed higher encapsulation efficiency (94.30%) than for the carriers individually (maltodextrin 91.5% and soybean protein isolate 89.83%)	
Yellow onions skins flavonoids extract		[67]
Carrier	Maltodextrin, pectin and whey proteins hydrolysates	
Conditions	Samples were frozen at −70 °C and then freeze-dried at −42 °C under a pressure of 0.1 mbar for 48 h	
Observations	Maltodextrin:pectin:whey proteins hydrolysates in a ratio of 2:1:0.4 resulted in the highest flavonoid encapsulation (66.46%)	
*Phoenix dactylifera* L pit polyphenols		[68]
Carrier	Gum Arabic and egg yolk protein	
Conditions	Samples were frozen at −80 °C for 12 h and then freeze-dried for 48 h	
Observations	Microparticles with a higher amount of egg yolk protein showed the highest encapsulation efficiency (99.75%); the lowest encapsulation efficiency was observed when gum Arabic and gum Arabic:egg yolk protein (3:1) were used (44.06% and 43.04%, respectively)	
*Elsholtzia ciliate* ethanolic extract		[69]
Carrier	Gum Arabic, maltodextrin, beta-maltodextrin, resistant-maltodextrin, skim milk and sodium caseinate	
Conditions	Samples were frozen at −80 °C for 24 h and then freeze-dried at −50 °C at 0.05 mbar for 24 h	
Observations	The highest encapsulation efficiency of total polyphenols content were observed with sodium caseinate (83.02%) while the lowest were with maltodextrin (21.17%)	
Blackberry juice polyphenols		[70]
Carrier	Apple fibers	
Conditions	Samples were frozen at −18 °C for 24 h and then freeze-dried under the following conditions: −55 °C—freezing temperature; −35–0 °C—temperature of sublimation; 0.220 mbar—vacuum level; 0–21 °C—isothermal desorption temperatures; 12 h—process duration	
Observations	Different amounts of apple fibers (1%, 2%, 4%, 6%, 8%, and 10%) were used for polyphenol encapsulation, and results showed the best adsorption of total polyphenols when 1% (1.82 g GAE/100 g) and 2% (1.79 g GAE/100 g) of fiber was used	
Blackberry juice polyphenols		[71]
Carrier	Citrus fibers	
Conditions	Samples were frozen at −18 °C for 24 h and then freeze-dried under the following conditions: −55 °C—freezing temperature; −35–0 °C—temperature of sublimation; 0.220 mbar—vacuum level; 0–21 °C—isothermal desorption temperatures; 12 h—process duration	
Observations	By increasing the amount of fiber above 1%, a decrease in the concentration of adsorbed polyphenols occurred; complexes with higher amounts of fiber (2% and 4%) had higher retention levels of polyphenols after eight months’ storage (70% and 79%, respectively)	
Raspberry juice polyphenols		[72]
Carrier	Cellulose	
Conditions	Samples were frozen at −18 °C for 24 h and then freeze-dried under the following conditions: −55 °C—freezing temperature; −35–0 °C—temperature of sublimation; 0.220 mbar—vacuum level; 0–21 °C—isothermal desorption temperatures; 12 h—process duration	
Observations	The complex with 2.5% of cellulose resulted in the highest concentration of polyphenols (2.43 g/kg for 15 min of complexation and 1.96 g/kg for 60 min of complexation); higher amounts of the carrier (5%, 7.5% and 10%) negatively affected polyphenols adsorption; the highest retention of polyphenols during storage was observed in powders with 5% and 7.5% of cellulose (from 90–100%)	

**Table 3:** Studies of spray-drying (SD) and freeze-drying (FD) applications for encapsulation of polyphenols from different sources.

Bioactive Material		Reference
Roselle (*Hibiscus sabdariffa* L.) anthocyanins		[16]
Carrier	Maltodextrin, gum Arabic, inulin and konjac	
Conditions	SD: 500 mL/h—flow rate; 150 °C—inlet temperature; 91 °C—outlet temperature FD: Not defined	
Observations	The freeze-dried sample with 100% konjac had the highest antioxidant content but with low encapsulation efficiency of 43.6% (anthocyanins located on the surface); a mixture of maltodextrin and gum Arabic provided powders with high antioxidant content and efficiency for both methods of drying (around 95%)	
Grape (*Vitis labrusca* var. Bordo) skin phenolic extract		[73]
Carrier	Gum Arabic, polydextrose, and partially hydrolyzed guar gum	
Conditions	SD: 0.60 L/h—flow rate; 140 °C—drying air temperature; 3.5 kg/cm^2^—air pressure; 40.5 L/h—air flow rate FD: Dispersions were frozen at −68 °C for 24 h and then freeze-dried at −57 °C for 48 h at vacuum pressure of less than 20 µm Hg	
Observations	Between these drying methods, there was no difference in polyphenol retention; the spray-drying method using 10% gum Arabic had the highest retention of phenolic compounds (25.03 mg GAE/g) as well as freeze-drying with 5% gum Arabic and 5% of polydextrose (24.57 mg GAE/g)	
Coffee grounds polyphenols extract		[2]
Carrier	Maltodextrin and gum Arabic	
Conditions	SD: 108 mL/h—flow rate; 100 °C—air inlet temperature; 75% (28 m^3^/h)—aspiration FD: The samples were previously frozen and then freeze-dried at −60 °C at 0.05 bar for 48 h	
Observations	In spray-drying, using maltodextrin as wall material achieved the best encapsulation of flavonoids (around 52%) while a combination of maltodextrin and gum Arabic was the best for encapsulation of total phenolic compounds (around 65%); In freeze-drying, 100% maltodextrin as wall material was the best for encapsulation of total polyphenols and flavonoids (62% and 73%, respectively)	
Model fruit juice (0.1% citrus pectin, 10% sucrose and 0.5% gallic acid)		[13]
Carrier	Maltodextrin and gum Arabic	
Conditions	SD: 72–144 mL/h—flow rate; 80–120 °C—inlet temperature; 600 L/h—nozzle air flow rate; 75% (28 m^3^/h)—aspiration FD: 300–500mTorr—chamber pressure; 0.3–0.7 °C—freezing rate; 16 ± 0.5 h—process duration	
Observations	The higher concentrations of gallic acid in freeze-dried samples were achieved with close to 100% gum Arabic and encapsulant concentration of 10–20% and with maltodextrin concentration of 80–100%; for spray-dried samples the best conditions also included 10–20% encapsulant concentration and maltodextrin:gum Arabic ratio of 50–80%	
Lemon by-product aqueous extract		[17]
Carrier	Maltodextrin, soybean protein and ȷ-carrageenan	
Conditions	SD: 125 °C—inlet temperature; 55 °C—maximum outlet temperature; 601 L/h—atomization air flow rate; 4 mL/min—liquid feed pump rate; 38 m^3^/h—main drying air flow rate; 70 °C—feed solution temperature; 70 mL—feed solution FD: Using liquid nitrogen, samples were initially frozen and then freeze-dried (48 h)	
Observations	Freeze-dried samples obtained with a combination of maltodextrin and soybean protein achieved the highest encapsulation productivity of total polyphenols content and total flavonoids content (74%); in spray-drying, the best encapsulation productivity for total polyphenols content (67%) was achieved with the same combination of wall materials as for freeze-drying, while for encapsulation productivity of total flavonoids content (58%), no statistical difference was observed between wall materials	
Acerola (*Malpighia emarginata* DC) pulp and residue		[74]
Carrier	Gum Arabic and maltodextrin mixture	
Conditions	SD: 1 mm—feed nozzle diameter; 4 m^3^/min—drying air flow rate; 0.36 L/h—feed rate; 30 L/min—compressed air flow; 3.5 kgf/cm^2^—air pressure; 170 °C—inlet temperature; 82 °C—outlet temperature FD: Samples were previously frozen at −18 °C for 48 h and then freeze-dried at −58.8 °C for 48 h at 0.42 a mbar vacuum	
Observations	Microencapsulation efficiency for total polyphenols content of freeze-dried samples of both pulp and residue was higher (around 68%) than for spray-dried samples; for microencapsulation efficiency of total flavonoids content, the best results (around 59%) were obtained for freeze-dried acerola residue	
Star fruit (*Averrhoa carambola*) pomace polyphenols		[41]
Carrier	Maltodextrin	
Conditions	SD: 185 °C—inlet temperature; 88 °C—outlet temperature; 6 mL/min—feed rate; 0.1 mm—nozzle size FD: Frozen samples (−40 °C) were freeze-dried at −55 °C for 24 h	
Observations	Freeze-dried encapsulates had a higher encapsulation efficiency (78–97%) than spray-dried ones (63–79%); an increase in maltodextrin concentration led to an increase in core polyphenols content	
Gallic acid		[33]
Carrier	Acid-hydrolyzed low dextrose equivalent potato starch	
Conditions	SD: 160 °C—inlet temperature; 75 °C—outlet temperature FD: Not defined	
Observations	Encapsulation efficiency for freeze-dried samples ranged from 70–84% and for spray-dried from 65–79% without statistically significant differences between methods	
Hydroxytyrosol		[3]
Carrier	β-cyclodextrin	
Conditions	SD: 100 °C—gas inlet temperature; 100 L/min—drying gas (air) flow rate; 0.5 mL/min—feed rate; 35 mbar—inside pressure; 100%—spray rate FD: Frozen samples were slowly dried at −50 °C under 0.06 mbar	
Observations	Spray-dried particles had a spherical and smooth surface with encapsulation efficiency of 84.4%; freeze-dried particles had an irregular shape and encapsulation efficiency was 89.6%	
Papaya pulp		[40]
Carrier	Maltodextrin	
Conditions	SD: 150 °C—inlet temperature; 4 m^3^/min—air flow; 3 kgf/cm^2^—air pressure; 0.4 L/h—feed flow FD: −62 °C—processing temperature; 6.11 mbar—vacuum degree; 48 h—process duration	
Observations	Freeze-dried products possessed lower retention of vanillic, ferulic, and caffeic acids (15.85 ng/g, under detection limit, and under detection limit, respectively) than spray-dried (30.73 ng/g, 11.26 ng/g, and 9.45 ng/g, respectively)	
*Moringa stenopetala* leaves extract		[75]
Carrier	Maltodextrin and high methoxyl pectin	
Conditions	SD: 0.5 mm—nozzle diameter; 485 mL/h—flow rate; 140 °C—inlet temperature; 78–81 °C—outlet temperature FD: Samples were frozen at −70 °C for 2 h and then freeze-dried for 72 h	
Observations	Total polyphenols content and total flavonoid content of freeze-dried samples were higher than in spray-dried ones, but encapsulation efficiency and storage stability were better in spray-dried samples; encapsulation efficiency for spray-dried powders with maltodextrin and maltodextrin/high methoxyl pectin was 83.52% and 87.93%, while for freeze-dried ones, it was 71.44% and 82.12%, respectively	
Cranberry juice		[76]
Carrier	Maltodextrin	
Conditions	SD: 50%—pump capacity; 35 m^3^/h—air flow FD: 0.03 mbar	
Observations	Powders obtained with sugar free cranberry juice and without maltodextrin had almost five times higher contents of polyphenols (6423 mg/kg—FD and 6433 mg/kg—SD) than powders with 15% maltodextrin and cranberry juice (961 mg/kg—FD and 807 mg/kg—SD); in samples with maltodextrin, *p*-coumaroyl-hexose concentration was higher when spray-drying was applied (180 mg/kg); considering anthocyanins, spray- and freeze-drying equally affected their retention	
Rose (*Rosa rugosa*) anthocyanins		[42]
Carrier	Gum Arabic and maltodextrin	
Conditions	SD: 170 °C—inlet temperature; 5 m^3^/min—drying airflow rate; 0.36 L/h—feed rate FD: −52 °C—processing temperature; 0.45 mbar—vacuum degree; 48 h—process duration	
Observations	The retention rate of total polyphenols content and anthocyanin content was 86% and 75.85% for spray-dried powder, and 91.44% and 95.12% for freeze-dried powder, respectively	
Lowbush *Vaccinium myrtillus* blueberry fruit juice		[24]
Carrier	Hydroxypropyl-β-cyclodextrin and maltodextrin	
Conditions	SD: 140 °C—inlet temperature; 70 °C—outlet temperature; 75%—air flow rate; 0.7 mm—nozzle diameter FD: Slowly freezing at −50 °C; pre-drying at 0.42 mbar and 30 °C; secondary drying—reducing pressure to 0.05 mbar and increasing temperature to 40 °C	
Observations	Spray-dried microparticles with maltodextrin and hydroxypropyl-β-cyclodextrin microcapsules had higher total polyphenols content and total anthocyanins content (around 1.65 g/100 g and 1.3 g/100 g, respectively) than freeze-dried microparticles with β-cyclodextrin (around 1.45 g/100 g and 1.1 g/100 g, respectively); total losses of anthocyanins and total polyphenols during drying were lower in the freeze-drying process	
Apple peel polyphenols		[77]
Carrier	Maltodextrin, gum Arabic, and whey protein concentrate	
Conditions	SD: 150 °C—inlet temperature; 50 °C—outlet temperature FD: Samples were frozen at −20 °C for 24 h and then freeze-dried at −45 °C under a pressure of less than 0.12 mbar for more than 48 h	
Observations	Freeze-dried samples homogenized by ultrasonication possessed higher encapsulation efficiency values for phenolic content (83.69%), flavonoid content (85.47%), and antioxidant activity (86.85%) compared to the spray-dried samples previously homogenized by ultra turrax (83.58%, 48.31% and 80.21%, respectively)	

**Table 4:** Selected studies on the incorporation of encapsulated polyphenols into food products.

Food Product	Source of Polyphenols	Wall Material	Encapsulation Technique	Major Findings	Reference
Biscuit	Italian black rice polyphenols	Maltodextrin Gum Arabic	Spray-drying Freeze-drying	Spray-dried encapsulates, added to biscuits, were the most stable during storage and were partially protected during the baking; enriched biscuits showed a higher content of polyphenols, anthocyanins and antioxidant activity than control biscuits	[84]
Biscuit	Cocoa hulls polyphenols	Maltodextrin Gum Arabic	Spray-drying	By using powder with maltodextrin, the most stable sample with unaffected total polyphenols content after baking was obtained	[85]
Cake	Sour cherry polyphenols	Maltodextrin Gum Arabic	Freeze-drying	The incorporation of encapsulated polyphenols didn’t impair the sensory or quality properties of cakes; a positive effect on hygroscopicity, baking stability, storage and digestibility was observed	[86]
Chocolate	Peanut skins polyphenols	Maltodextrin	Spray-drying	Antioxidant activity increased after the addition of encapsulates; with 9% of additives antioxidant activity was similar to dark chocolate while flavor was similar to milk chocolate	[87]
Jelly	Barberry polyphenols	Maltodextrin Gum Arabic Gelatin	Spray-drying	Gum Arabic/maltodextrin was the best wall material; jelly with 7% of powder showed better consumer acceptability than commercial jelly; antioxidant activity was increased	[88]

## Data Availability

Not applicable.
